# Hypertension Research in Pakistan: A Scientometric Analysis of Two Decades (2003-2022)

**DOI:** 10.7759/cureus.59769

**Published:** 2024-05-06

**Authors:** Fahad Anwer, Ahmad Azam Malik

**Affiliations:** 1 Department of Family and Community Medicine, Faculty of Medicine in Rabigh, King Abdulaziz University, Jeddah, SAU

**Keywords:** web of science, scientometric analysis, pakistan, research, hypertension

## Abstract

Hypertension is a highly prevalent chronic disease that leads to a significant number of deaths and disabilities as a consequence of cardiovascular complications. However, there is insufficient information regarding research trends and performance assessments from South Asian countries, including Pakistan. In this review, we analyzed research data related to hypertension from Pakistan over the last 20 years (2003-2022). We used the Web of Science (WoS) database to retrieve research data related to hypertension from Pakistan, and then applied scientometric analysis using the “R-Bibliometrix” package. An extensive range of indicators was studied to determine the quality and quantity of these hypertension-related publications. A total of 4,008 research articles from 891 sources were extracted through WoS over the last 20 years (2003-2022). There was a continuous growth in the number of research articles, with relatively more increase observed from 2012, and maximum output in 2021. Among 16,855 authors contributing from 67 countries, three authors had >50 publications and five had >1,500 citations. The country’s leading affiliation was the Aga Khan University which showed networking trends with international institutes while the other national universities restricted their institutional partnerships to the provincial or city level. The Higher Education Commission was the only local sponsoring institute among the top funding sources. Pakistan Journal of Medical Sciences was the leading and most consistent source, whereas hypertension, diabetes mellitus, and obesity were the most frequently used keywords. This review provides a comprehensive account of hypertension-related research productivity from Pakistan. Some characteristic trends were detected for top authors’ contributions, impact, productivity, international collaborations, funding sources, and institutional affiliations. Particularly, the funding sources and collaboration patterns of corresponding authors along with their affiliated institutes showed striking results. These findings can be very helpful for the relevant stakeholders in the accurate interpretation of trends and performance of hypertension-related research work from the region.

## Introduction and background

Hypertension is regarded as a key contributing risk factor for chronic renal disease, cardiovascular disease, and stroke, as well as a major source of the global burden of non-communicable diseases [1]. As scientific research has proven hypertension can gradually harm target organs without causing symptoms, it is known as a silent killer [2].

The World Health Organization (WHO) estimates that 1.28 billion people globally, aged 30-79 years, suffer from hypertension. Less than half of these adults with hypertension are diagnosed and receive treatment, whereas an estimated 46% do not know that they have the disease [3]. The global rate of hypertension did not significantly alter between 1990 and 2019, although low- and middle-income countries carry the bulk of the disease burden at present. Overall, 82% of the world’s hypertensive population, or over one billion individuals, resided in low- and middle-income nations in 2019. Moreover, according to the American College of Cardiology in 2018, hypertension prevalence ranged from 4% to 78% worldwide in 71 countries [4]. As the prevalence of hypertension is rising, there is a corresponding rise in the incidence of cardiovascular illnesses and their consequences, which can put a tremendous strain on healthcare systems [5]. Furthermore, there are financial ramifications for national drug plans because of the mounting evidence that the majority of people with hypertension need two or more drugs to control their blood pressure [6].

Despite a steady global age-standardized prevalence, the number of people with hypertension aged 30-79 years more than doubled between 1990 and 2019. In 1990, there were 331 (95% credible interval = 306-359) million women and 317 (292-344) million men with hypertension, but by 2019, this number had increased to 626 (584-668) million women and 652 (604-698) million men. Worldwide, 59% (55-62) of women and 49% (46-52) of men with hypertension reported having received a previous diagnosis in 2019. Moreover, treatment was received by 38% (35-41) of men and 47% (43-51) of women [7].

The prevalence of hypertension among adults worldwide is currently 25%, and by 2025, it is expected to reach 29% [8]. Relative to high-income countries (28.5%), the prevalence of hypertension was found to be 31.5% in low- and middle-income countries based on a systematic review of population-based research data from 90 countries [9]. In addition to the rising prevalence of hypertension in developing countries, cardiovascular disease (including hypertension) tends to occur at a younger age and, consequently, accounts for around 70% of mortality linked to high blood pressure [8].

A systematic review of data from SAARC (South Asian Association of Regional Cooperation) countries found a high prevalence of hypertension in South Asian countries. Pakistan was found to have a 25% prevalence [10]. Nearly 18.9% of Pakistanis over the age of 15 years have hypertension, according to the most recent population-based National Health Survey of Pakistan. This percentage is higher in men than women and in urban areas compared to rural areas. [11]. Studies have been conducted to ascertain the prevalence of hypertension periodically in several Pakistani provinces and cities, despite a lack of population-based statistics on the chronic condition. A recent study on hypertension in Pakistan revealed that there is a high prevalence of non-communicable diseases, including hypertension, particularly in the Punjab and Sindh provinces. Hypertension prevalence was found to be 46.2% in the National Diabetes Survey of Pakistan 2016-2017, a sizable community-based epidemiological study that included 10,834 participants across all four provinces [12]. According to other small-scale urban studies, the prevalence of hypertension ranged from 15% to 29%. [13,14]. A South Asian study performed on the population of three cities (Chennai, Delhi, and Karachi) found the age-adjusted prevalence of hypertension of 24% in men and 28% in women in Karachi [15].

A relatively new branch of information theory called scientometric analysis uses quantitative methods for evaluating the characteristics and behaviors of previously documented research. As a result, studies now employ objective data analysis and quantitative measurements. This novel method of analysis yields objective statistics on research output within a disease area, medical specialty, or discipline. Temporal trends can be determined by comparing nations, institutions, and disciplines using plausible indicators [16]. It can be very useful in generating relevant data to help countries enhance their research policies. Over the past two decades, there has been a significant rise in the research output related to this domain [17].

Despite the high prevalence of hypertension in Pakistan, as evident from the above studies, the research productivity from the country targeting this significant chronic disease remains largely unidentified. Evaluating the existing status of medical research on this growing health problem should be a high priority as this will help in structuring better plans for investments in the future. This review aimed to evaluate the advancements in hypertension-related research from Pakistan that have been indexed in the Web of Science (WoS) over the last 20 years (2003-2022) utilizing several extensively used scientometric markers. Policymakers may find this evaluation of research performance and trends helpful in allocating resources and making informed decisions.

## Review

Methodology

In this review, we performed quantitative analysis using scientometric tools to explore various trends and patterns in the hypertension-related research output from Pakistan. According to World Bank figures for the year 2022, Pakistan’s population is approximately 230 million, placing it in the low-middle-income category. The gross domestic product of the nation is $348,26 billion, with 3% allocated for healthcare [18]. Among the wide range of available databases, such as Scopus, EBSCO, ScienceDirect, ProQuest, and PubMed, we chose the most useful and widely used database, WoS, to extract the required data using suitable criteria, search subjects, and identified keywords from literature. WoS is managed by Clarivate Analytics (previously Thomson Reuters). It is regarded as the most accurate, thorough, and richly indexed source for scientific exploration. Additionally, it is thought to be more suitable for assessing the research productivity of various authors, organizations, or geographical areas [19]. It includes more than a billion searchable cited references as well as a search option across key search databases, disciplines, and document categories [20]. Information was accessed from WoS using King Abdulaziz University’s digital resources and online library. To ensure the integrity of data during the primary extraction and subsequent processing stages, this review employed scientometric methodologies to evaluate every article that was published in WoS between 2003 and 2022 and covered hypertension explicitly.

We used the following search strategy: TS= (Hypertens* OR HTN OR High Blood Pressure), filtered by CU=Pakistan, period (2003-2022), and Indexes (SCI Expanded, SSCI, A&HCI, CPCI-S, CPCI-SSH, ESCI). The utilization of the term “hypertens∗” indicates that all pertinent derivatives of the term “hypertension,” such as “hypertension” and “hypertensive,” were likewise evaluated and that relevant records were obtained. The search was conducted by two reviewers on a single day (November 28, 2023) to eliminate contradictions. After identification, the extracted record’s content was screened for inclusion in the analysis. We included all articles from Pakistan focussing on hypertension in the English language published between 2003 and 2022. Articles published after December 31, 2022 (344) were excluded from the study due to incomplete data in WoS for the year 2023 at the time of data extraction. Records other than documents and non-English-language articles were also excluded from the study.

Following the retrieval of data from WoS into plain text files, scientometric analysis was performed at the author, source, and document levels using the “R-Bibliometrix” tool [21]. A variety of scientometric computations, including journals, publication years, authors, citation reports, institutions, and countries/regions, were employed to evaluate the data related to extracted documents.

Results

When explored for this review’s scope globally, the United States, China, and Japan were leading contributors with 31.8%, 13.6%, and 7.7%, respectively. Contribution from Pakistan was 4,008 articles, representing around 0.91% of the global productivity and ranking 31st among all.

For a detailed analysis of this review, 4,008 research publications were retrieved from 891 sources between 2003 and 2022. Of those, 2,707 (86.5%) were published from 2013 to 2022. The research output increased yearly by 21.74%. The figures reveal that 5.98 documents were produced annually on average. The mean citation score per document was 17.43, while the total number of references used was 91,690. The overall number of authors participating in research was 16,855, of whom 40 had single-authored publications. There were 50 single-authored documents. The overall contribution from international co-authors was around 34%. The total number of keywords used by authors was 8,405 (Table 1).

**Table 1:** Summary table.

Description	Results
Main information about the data	
Timespan	2003–2022
Sources (journals, books, etc.)	891
Documents	4,008
Annual growth rate (%)	21.74
Document average age	5.98
Average citations per doc	17.43
References	91,690
Document contents	
Keywords plus (ID)	5,274
Author’s keywords (DE)	8,405
Authors	
Authors	16,855
Authors of single-authored docs	40
Authors collaboration	
Single-authored docs	50
Co-authors per doc	9.19
International co-authorships (%)	34.13

**Figure 1:**
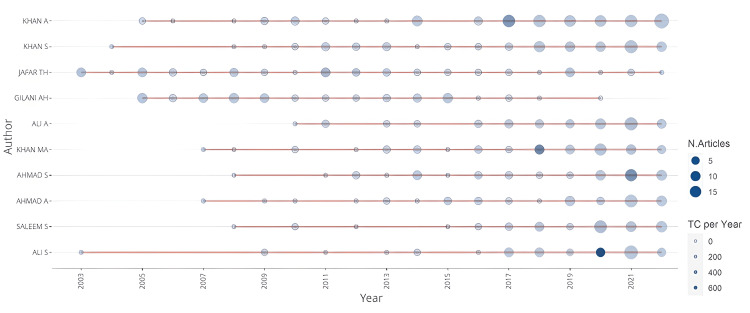
The 10 most productive authors with authors’ impact.

Figure 1 shows the 10 most productive authors along with the authors’ impact. Findings show that Khan A (96 publications), Khan S (63 publications), and Jafar TH (52 publications) were the top three authors, with the latter being the most consistent contributor to hypertension research productivity throughout the 20 years. Jafar TH and Ali S had the longest timeline (20 years) of research productivity. Among these top authors, Jafar TH achieved the maximum number of total citations (5,295). The other two authors achieving more than 3,000 citations were Ali S and Khan A. Overall, five authors achieved more than 1,500 total citations. When analyzed for H-index, two authors achieved a score of >20. Jafar TH achieved the highest H-index score (28).

Table 2 shows the top 10 countries according to the highest contributions from corresponding authors. Of all the articles, 3,191 (79.6%) had corresponding authors from Pakistan, followed by 161, 104, and 76 articles from the United States, China, and the United Kingdom, respectively. Around 82% of Pakistani corresponding authors had single-country publications.

**Table 2:** The top 10 countries according to most contributions from corresponding authors. SCP = single country publications; MCP = multiple country publications

Country	Articles	SCP	MCP	MCP ratio
Pakistan	3,191	2,622	569	0.178
United States	161	1	160	0.993
China	104	0	104	1
United Kingdom	76	1	75	0.986
Saudi Arabia	66	4	62	0.939
Malaysia	61	0	61	1
India	55	3	52	0.945
Canada	49	1	48	0.979
Japan	19	0	19	1
Singapore	16	0	16	1

Table 3 shows the most frequent affiliations and funding sources. Among more than 5,800 affiliated institutes contributing to producing 4,008 articles related to hypertension, Aga Khan University was the most productive affiliated institute with 541 articles. Among other affiliations in the top 10 list, only three had ≥150 published articles, namely, Dow University of Health Sciences, Comsats University Islamabad, and Karachi University contributing to 292, 153, and 150 articles, respectively. Among the funding sources, the Higher Education Commission of Pakistan was the leading source contributing to 132 publications, followed by the United States National Institutes of Health and the United States Department of Health and Human Services funding 92 publications each.

**Table 3:** The top 10 most frequent affiliations and funding sources.

Affiliations	Frequency	Funding organizations	Frequency
Aga Khan University	541	Higher Education Commission, Pakistan	132
Dow University of Health Sciences	292	National Institutes of Health, USA	92
Comsats University Islamabad	153	United States Department of Health and Human Services	92
University of Karachi	150	UK Research Innovation (UKRI)	61
Quaid e Azam University	136	Wellcome Trust	56
University of Punjab	110	Medical Research Council, UK (MRC)	55
National University of Sciences Technology	106	Canadian Institutes of Health Research (CIHR)	42
King Saud University	100	National Institutes of Health Research (NIHR)	42
King Edward Med University	99	Consultative Group on International Agricultural Research (CGIAR)	38
University of London	99	National Natural Science Foundation of China (NNFC)	36

**Figure 2:**
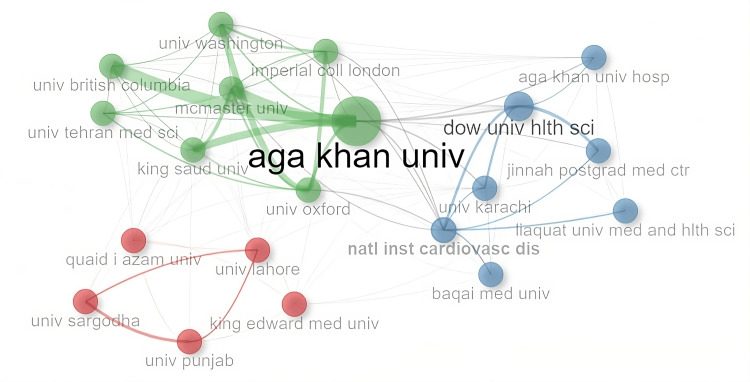
The top 20 affiliations collaboration network with major clusters.

In the collaboration network of the top 20 affiliations, as shown in Figure 2, three clusters were observed. The largest cluster (green) consisted of Aga Khan University as the only national institute with all others being international universities and institutions from countries such as the United Kingdom, the United States, and Saudi Arabia, among others. The other large cluster (blue) was formed by Dow University of Health Sciences, Karachi, and other institutes and Universities of Karachi such as the National Institute of Cardiovascular Diseases and Baqai Medical University as significant contributors. The third minor cluster (red) was formed by five universities in the Punjab province of the country.

**Figure 3:**
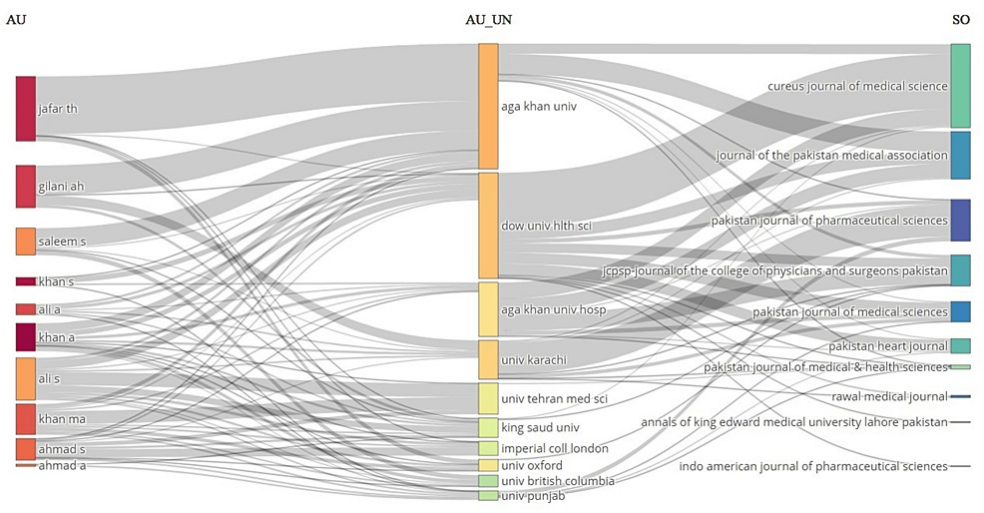
Three-field plot for the top 10 most productive affiliations, authors, and sources. AU = authors; AU_UN = author’s affiliations; SO = sources

A three-field plot for the top 10 most productive affiliations, authors, and sources in Figure 3 shows that the three leading authors were Jafar TH, Gilani AH, and Ali S. Aga Khan University and Dow University of Health Sciences were the leading institutes for research productivity. The top sources observed were the Cureus Journal of Medical Science, the Journal of Pakistan Medical Association (JPMA), the Pakistan Journal of Pharmaceutical Sciences, and the Journal of College of Physicians and Surgeons of Pakistan (JCPSP). These journals gave the institutional platform to many contributing authors, with most of their work being published in the Cureus Journal of Medical Science. The University of Karachi was the major contributor to publications in the Pakistan Journal of Pharmaceutical Sciences. The majority of publications of both Jafar TH and Gilani AH were under the affiliation of Aga Khan University. The research productivity from Aga Khan University was relatively higher in the JPMA, followed by the Cureus Journal of Medical Science, while a reasonable proportion of research articles from Dow University of Health Sciences was published in the Cureus Journal of Medical Science.

Table 4 shows the list of most highly cited research articles according to the achievement of most local citations. Jafar TH was the author of the top four highly cited articles in the local context. Two articles had more than 50 local citations. In this list, only one article had global citations >1,000, belonging to Chow CK, and published in the Journal of American Medical Association (JAMA) in 2013. Moreover, research by Joshi P, published in JAMA in 2007 achieved >500 global citations. Six of these top articles were published in Q1 journals. Seven of these articles were based on cross-sectional study designs.

**Table 4:** Top 10 highly cited documents and their impact. IF = impact factor; JIF = journal impact factor

Authors	Study type	IF/JIF quartile	Year	Local citations	Global citations
Jafar et al. (2003) [22]	Cross-sectional	4.9/Q2	2003	60	116
Jafar et al. (2005) [23]	Cross-sectional	3.2/Q3	2005	54	128
Jafar et al. (2006) [24]	Cross-sectional	14.6/Q1	2006	44	208
Jafar et al. (2008) [25]	Cross-sectional	5.7/Q1	2008	32	86
Chow et al. (2013) [26]	Cross-sectional	120.7/Q1	2013	26	1212
Joshi et al. (2007) [27]	Case-control	120.7/Q1	2007	24	594
Basit et al. (2018) [28]	Cross-sectional	2.9/Q2	2018	23	123
Ghayur et al. (2005) [29]	Randomized controlled	1.4/Q4	2005	22	135
Jafar et al. (2009) [30]	Cluster randomized controlled	39.2/Q1	2009	22	127
Shafi et al. (2017) [31]	Cross-sectional	7.3/Q1	2017	22	46

**Figure 4:**
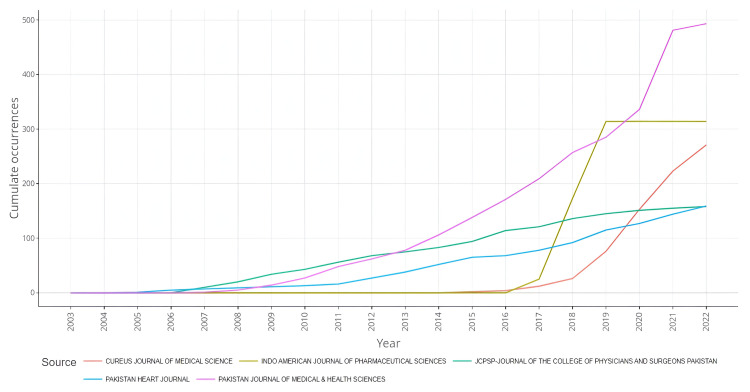
Year-wise growth of the five most productive sources.

Figure 4 shows the year-wise growth of the five most productive sources. The Pakistan Journal of Medical and Health Sciences was the most productive journal, showing a steady rise in publications between 2008 and 2020, and thereafter, a sharp rise in publications can be seen, reaching close to 500 publications in 2022. The second-most productive journal, the Indo-American Journal of Pharmaceutical Sciences, displayed a significant spike in publications between 2017 and 2019, which remained steady thereafter. The Cureus Journal of Medical Science also showed increasing growth in the number of publications from 2017. JCPSP and the Pakistan Journal of Medical and Health Science mostly showed a steady increase in publications.

**Figure 5:**
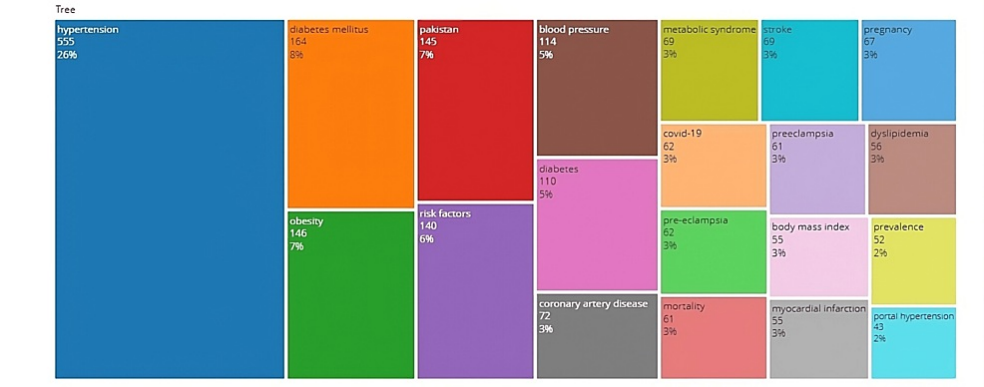
The top 20 most frequent keywords.

**Figure 6:**
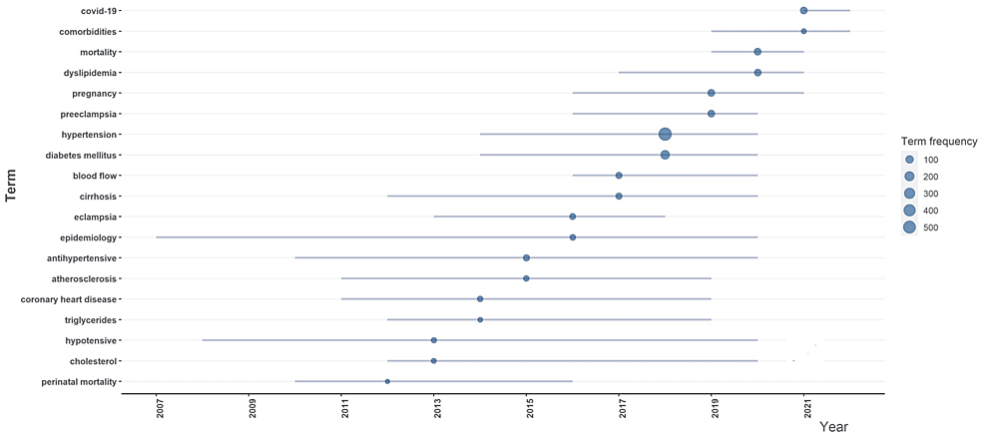
Trend of keywords by year with term frequency.

The Treemap in Figure 5 shows “hypertension,” “diabetes mellitus,” and “obesity” as the most frequently used keywords, while Figure 6 displays the annual trends of keywords with “hypertension” and “diabetes mellitus” as the most frequent keywords while “epidemiology” and “hypotensive” having the longest timelines. After 2020, the most frequent search words were COVID-19 and comorbidities.

Discussion

The value of health and the necessity of evidence-based health practices have been widely recognized in recent years, leading to a significant rise in the importance of healthcare research. Hypertension research is also continuing forward, and its landscape is continuously evolving. The data related to trends and performance of hypertension research is scarce from the South Asia region, including Pakistan. To our knowledge, this is the first bibliometric review analyzing the research data for hypertension in Pakistan. This review provides an in-depth assessment of publications on hypertension from Pakistan. This will assist in focusing on key areas for future research in hypertension and guiding the allocation of the already limited resources of the country toward the correct channels. In addition to academicians, physicians, and researchers, the findings of this review will also benefit policymakers and other important stakeholders of the region, including Pakistan, to understand the latest trends and direct possible future research planning, especially concerning providing more funding from local organizations for hypertension research and supporting international collaborations by local authors and their affiliated institutes.

The annual research productivity related to hypertension increased gradually over time, with a sharp increase in publications seen after 2107 and reaching its peak in 2021 with more than 550 publications. Surprisingly, this pattern is quite similar to the research trends observed in a study analyzing trends in diabetes research in Pakistan [32]. There may be a few factors contributing to this trend. One of them could be the growing interest of researchers in studying hypertension and its management in the presence of other comorbidities, especially cardiovascular risk factors, and searching for better drug treatment options. In recent years, there has been a considerable increase in the academic institutes in Pakistan which have been emphasizing more on research. They have established it as a mandatory criterion for their faculty members to have enough research publications for academic promotions. This incentive might have compelled the medical faculty to enhance their research productivity related to hypertension as well. Moreover, we observed a surge in the output of published medical journals in the country. Many of these journals established their credibility and acquired rankings in the WoS which could have led to the recent interest of Pakistani researchers to publish in these local journals.

The author showing the highest productivity in hypertension research was Jafar TH by a considerable margin, with more than 5,000 citations and a relatively high H-index of 28. The most probable reason for this observation is that she had the longest period of publications in which she consistently churned high-impact articles annually. Another major factor is that she had five publications with the highest impact in the list of top 10 highly cited articles raising her as the most impactful author among all. Among the highly cited countries, the United States was at the top, followed by Pakistan, implying that these countries had the greatest impact on the country’s research productivity.

According to this review, the number of corresponding authors from Pakistan was around 80% of the total author pool contributing to hypertension research, followed by authors from the United States, China, and the United Kingdom. This proportion is similar and slightly more than that observed in a similar scientometric study from Saudi Arabia on diabetes mellitus [33] which showed around two-thirds of corresponding authors contributing locally. Pakistani corresponding authors produced single-country publications, emphasizing the importance of collaborating more internationally to increase the productivity of multiple-country publications, particularly by including neighboring countries with similar settings.

Notably, eight out of the top 10 institutions contributing to hypertension research belonged to Pakistan. However, among the funding organizations, the Higher Education Commission, despite being the foremost institute for funding the country’s hypertension research projects, was the only local funding body in the list of top funding organizations. These findings suggest that the lack of local funding sources in Pakistan could have limited the volume of achievable research. Limited resources could also have impeded the development of a local culture of high-quality research, resulting from a dearth of skilled and motivated researchers, scarcity of cutting-edge research facilities, insufficient financing, and fewer locally published high-quality publications.

In the affiliation’s collaboration network, the largest cluster including Aga Khan University showed partnerships with international institutes and none with any local university. These institutions were from the United Kingdom, Canada, and the United States. On the other hand, the second cluster including Dow University of Health Sciences only showed collaboration with other universities and medical institutes in Karachi. This is also a unique observation of affiliations limited to a city only. The third and smallest group of affiliations was demonstrated by five universities in Punjab province, suggesting an example of collaboration limited to the provincial level. Universities and institutions other than Aga Khan University should make efforts to enhance their collaborations with foreign universities.

Among the documents related to hypertension, only two articles attained a local citation score of >50. Both belonged to Jafar TH and were published in the Journal of Hypertension and the American Heart Journal [22,23]. Both were based on population-based cross-sectional surveys and explored hypertension in different ethnic groups of Pakistan. Analyzing global citations, one article achieved a score of >1,000 belonging to Chow CK [26], while another article authored by Joshi P had a global citation score of >500 [27]. Both articles were published in JAMA. The most appreciable strength of the study by Chow CK [26] was that it evaluated various aspects of hypertension while comparing them in countries grouped by income. Presumably, this multi-centric and multi-country approach is a major factor in achieving this high number of citations.

It was observed that the highest number of hypertension-related articles was published by Lancet, with nearly 2,400 research articles. It was followed by Circulation and the New England Journal of Medicine, with each contributing more than 1,500 publications. Perplexingly, none of these journals have a mention among the journals contributing to the highly cited documents despite playing a prominent role in contributing to the overall number of research publications.

The Pakistan Journal of Medical and Health Sciences was the leading source, with a consistent increase in its research output since 2007. Interestingly, the Indo-American Journal of Pharmaceutical Sciences, which was the second-most productive source, only started publishing in 2017 and showed a steep rise in publications till 2019, before following a very steady course probably because of a slight decline in the Journal’s ranking in indexing databases. Another noticeable observation was the curve displayed by the Cureus Journal of Medical Science, showing a progressive increase in publications since 2017. This trend is very similar to its increase in research productivity shown in a similar analysis performed on hypertension research output from Saudi Arabia [34]. The main factor behind this trend seems to be the indexing status of the Journal in the ISI master Journal list. Journals achieving ranking in ISI, presumably, show a sudden increase in research productivity after their status upgrade.

Hypertension, diabetes mellitus, and obesity were the most frequently used keywords. The other notable observations were the search keyword categories in different interdisciplinary aspects related to HTN. Most keyword search choices were related to hypertension itself or its comorbid conditions or complications. This suggests that the researchers are not studying hypertension as an exclusive entity but analyzing it in the context of different clinical paradigms. The major theme of interest for the researchers was cardiovascular disease and its prevention. It also emphasizes the altering shift of researchers’ priorities in selecting their study areas related to hypertension. Examining the recent trend of keywords, experts have been more intrigued to study how COVID-19 infection may affect the clinical parameters of hypertension in the last two years. This is not unexpected as this viral infection (pandemic) became the focus of all medical fraternities during this period.

There are some limitations to this review. The analysis was planned on the data extracted from WoS only. This approach may have curtailed the applicability of the review findings to global research output on hypertension. Limitations of WoS inclusive of the ongoing modifications and updates may produce varying research data for analysis based on the search data and period. Moreover, scarce data availability in the context of this review further constrained the comparison with previous decades and from other sources. However, the results of the analysis of the most frequently used keywords enhance the search strategy’s validity.

## Conclusions

This scientometric review on hypertension in Pakistan has yielded a few significant findings that may have consequences for future research-related developments. There has been an increasing trend in hypertension-related research productivity from Pakistan, particularly in the last three years, but the collaborative trends between affiliated institutes and corresponding authors need to be sustained and expanded beyond the borders, with foreign colleges and universities. There is a growing need for more funding contributions toward hypertension research from institutes within Pakistan. As there is insufficient information to assess the adequacy of hypertension research, more related studies are required in Pakistan and other South Asian countries. Further research will make it more feasible to investigate all crucial relevant facets of research productivity on this major chronic illness and create a more effective hypertension research plan specific to regional perspectives.
